# Aging-Dependent Genetic Effects Associated to ADHD Predict Longitudinal Changes of Ventricular Volumes in Adulthood

**DOI:** 10.3389/fpsyt.2020.00574

**Published:** 2020-06-29

**Authors:** Natalia Vilor-Tejedor, Mohammad Arfan Ikram, Gennady Roshchupkin, Elisabeth J. Vinke, Meike W. Vernooij, Hieab H. H. Adams

**Affiliations:** ^1^ Centre for Genomic Regulation (CRG), The Barcelona Institute for Science and Technology, Barcelona, Spain; ^2^ BarcelonaBeta Brain Research Center (BBRC), Pasqual Maragall Foundation, Barcelona, Spain; ^3^ Department of Clinical Genetics, Erasmus Medical Center, Rotterdam, Netherlands; ^4^ Universitat Pompeu Fabra (UPF), Barcelona, Spain; ^5^ Department of Epidemiology, Erasmus Medical Center, Rotterdam, Netherlands; ^6^ Department of Radiology and Nuclear Medicine, Erasmus Medical Center, Rotterdam, Netherlands; ^7^ Department of Neurology, Erasmus Medical Center, Rotterdam, Netherlands; ^8^ Department of Medical Informatics, Erasmus Medical Center, Rotterdam, Netherlands

**Keywords:** adulthood, aging, brain atrophy, brain trajectories, neurogenetics, rs212178, ventricle size

## Abstract

**Background:**

Attention-Deficit/Hyperactivity Disorder (ADHD) is a childhood-onset disorder that can persist into adult life. Most genetic studies have focused on investigating biological mechanisms of ADHD during childhood. However, little is known about whether genetic variants associated with ADHD influence structural brain changes throughout adulthood.

**Methods:**

Participant of the study were drawn from a population-based sample of 3,220 healthy individuals drawn from the Rotterdam Study, with at least two magnetic resonance imaging (MRI)-scans (8,468 scans) obtained every 3–4 years. We investigate associations of genetic single nucleotide polymorphisms (SNPs) that have previously been identified in genome-wide association studies for ADHD, and trajectories of global and subcortical brain structures in an adult population (aged 50 years and older), acquired through MRI. We also evaluated the existence of age-dependent effects of these genetic variants on trajectories of brain structures. These analyses were reproduced among individuals 70 years of age or older to further explore aging-dependent mechanisms. We additionally tested baseline associations using the first MRI-scan of the 3,220 individuals.

**Results:**

We observed significant age-dependent effects on the rs212178 in trajectories of ventricular size (lateral ventricles, P= 4E-05; inferior lateral ventricles, P=3.8E-03; third ventricle, P=2.5E-03; fourth ventricle, P=5.5E-03). Specifically, carriers of the G allele, which was reported as protective for ADHD, had a smaller increase of ventricular size compared with homozygotes for the A allele in elder stages. Post hoc analysis on the subset of individuals older than 70 years of age reinforced these results (lateral ventricles, P=7.3E-05). In addition, the rs4916723, and the rs281324 displayed nominal significant age-dependent effects in trajectories of the amygdala volume (P=1.4E-03), and caudate volume (P=1.8E-03), respectively.

**Conclusions:**

To the best of our knowledge, this is the first study suggesting the involvement of protective genetic variants for ADHD on prevention of brain atrophy during adulthood.

## Introduction

Attention-Deficit/Hyperactivity Disorder (ADHD) is a childhood neurodevelopmental disorder with an estimated worldwide prevalence of 5.2% (1–3). Although it is most common in children, recent work suggests that for some individuals, ADHD first emerges in adulthood (4), and one-sixth of individuals with a childhood diagnosis continue to meet clinical criteria for ADHD in adulthood (5–7).

It is challenging to characterize the determinants of the persistence and/or occurrence of ADHD through adulthood because the normal aging process mimics some classic ADHD symptoms. However, it is well established that genetic factors explain a large part of the individual differences in the vulnerability for ADHD (75%–90% in children, 30%–50% in adults) (8–10).

Knowing which genetic variants are associated with ADHD set the interest to understand how they could act on the brain to bring about ADHD. For instance, given that structural magnetic resonance imaging (MRI) markers maybe even better suited as intermediate phenotypes than ADHD symptoms, and these measures generally show strong test-retest reliability (11–13), an increasing number of studies have attempted to examine whether genetic variants for ADHD could have distinct effects on the brain, thereby elucidating the causal pathway to disease (14–17).

Furthermore, it has been suggested that the genetic basis of the disorder may vary depending on the age (18). In line with these results, recent findings showed that genetic factors implicated in ADHD during childhood (cADHD) play different roles in adult ADHD (aADHD), which in turn, may lead to a different genetic influences across the development of these symptoms (19–22). Indeed, family genetic studies in clinical samples hinted that there may be a higher familial liability for aADHD compared with cADHD (23), which supports that aADHD symptoms may have stronger genetic liability. Therefore, examining the genetic basis of aADHD symptoms can offer reliable etiological information.

Moreover, whilst longitudinal studies are essential in characterizing differences on neuroanatomical trajectories attributed to genetic variants (20), most of ADHD studies have focused exclusively on cross-sectional associations (24–26). Also, the lack of age-proper clinical measures has constrained progress in the field.

The current study aimed to examine the associations between genome-wide significant SNPs reported in the latest genome-wide association (GWAS) meta-analysis for ADHD (27), and trajectories of global and brain subcortical structures in a sample of non-diagnosed individuals, to provide a more precise understanding of how genetic markers associated with ADHD shape brain structural variations, and how age-dependent genetic effects influence regional brain changes throughout adulthood. Moreover, due to the polygenic architecture of ADHD, where common risk alleles have small effect sizes, we also inspected associations between a composite genetic risk score (GRS) and brain structural trajectories.

## Materials and Methods

### Study Population

The study sample was drawn from the Rotterdam Study, an ongoing population-based cohort study in Netherlands, which currently consists of 14,926 individuals aged 45 years or more at baseline (28). At study entry, and every 3–4 years, participants visited the dedicated research center for extensive investigations. Simultaneously, electronic data linkage with general practitioners recorded incident events or diagnoses. A total of 5,430 individuals were scanned through magnetic resonance imaging (MRI) and were eligible for this study. Individuals with only a single MRI scan, incomplete MRI-acquisitions, scans with artifacts and dementia or stroke were excluded. The Rotterdam Study has been approved by the Medical Ethics Committee of the Erasmus MC and by the Ministry of Health, Welfare and Sport of Netherlands, implementing the Wet Bevolkingsonderzoek: ERGO (Population Studies Act: Rotterdam Study). All participants provided written informed consent to participate in the study and to obtain information from their treating physicians. Data on ADHD diagnosis or symptomatology was not collected because of the study’s focus on late-onset diseases.

### Image Acquisition, Processing, and Selection

Magnetic Resonance Imaging scanning was done on a 1.5-T MRI scanner (Sigma Excite II; General Electric Healthcare, Milwaukee, WI, USA). Brain MRI scans included a high-resolution 3D T1-weighted fast radio frequency spoiled gradient-recalled acquisition in steady-state with an inversion recovery prepulse (FASTSPGR-IR) sequence (29). Sequence parameters were TR = 700 ms, TE = 14 ms, matrix side of 192 × 256 and flip angle = 70 with a voxel size of 1 × 1 × 1 mm.  All participants were imaged on the same scanner with fixed protocol and imaging parameters. A total of 12,174 brain MR-scans have been obtained in over 5,430 individuals, as of July 2015. The T1-weighted MRI scans were used to calculate global and subcortical structures and thickness of the cerebral cortex using a standard model-based automated procedure on Freesurfer (30) (version 5.1) image analysis suite. Quality control included the removal of outliers, as well as unusual brain volume values. We additionally excluded individuals with dementia and/or stroke. The brain measures included in the analyses were: cerebral white matter, cerebral grey matter, total intracranial volume, lateral ventricles, inferior lateral ventricles, cerebellum white matter, cerebellum cortex, thalamus, corpus callosum, caudate, putamen, pallidum, hippocampus, amygdala, accumbens area, third ventricle, fourth ventricle, and cerebrospinal fluid. The value of the brain measures used as the outcomes of the study was calculated as the average of the regional value of each hemisphere (mm3). From the total sample of individuals included in the study (N=5,430), a total of 3,220 have at least two scan measurements, 1,887 have at least three scan measurements, and 141 have four scan measurements. Further details of the MRI protocol, can be found in (29).

### Genotyping Acquisition and Genetic Variant Selection

The Illumina 550K, 550K duo, and 610K arrays were used for genotyping samples with a call rate below 97.5%, gender mismatch, excess autosomal heterozygosity (>0.336), duplicates or family relations and ethnic outliers were excluded. Genetic variants were filtered by Hardy-Weinberg equilibrium (P<10^-6^), allele frequency (excluding minor allele frequency (MAF < 0.001) and SNP call rate with a minimum of 98%. Genotypes were imputed using MACH/minimac software to the 1000 Genomes phase I version three-reference panel (all populations). From the imputed data (HRC version 1.1), we selected eight SNPs associated with ADHD at a genome-wide threshold of significance (P<10^-8^) [**Table SM1**] (27). Four genetic variants were not included because were not available in the HRC imputations, nor were there are any variants in linkage disequilibrium, likely because the original ADHD GWAS used a custom genotyping array for psychiatric disorders. Furthermore, we constructed a genetic risk score (GRS) by multiplying the number of risk alleles by their reported odds ratio (after natural logarithm transformation) for the disease and summing this weighted allele score of each variant up into a disease risk score for ADHD.

### Statistical Analysis

#### Longitudinal Models

**Figure 1:**
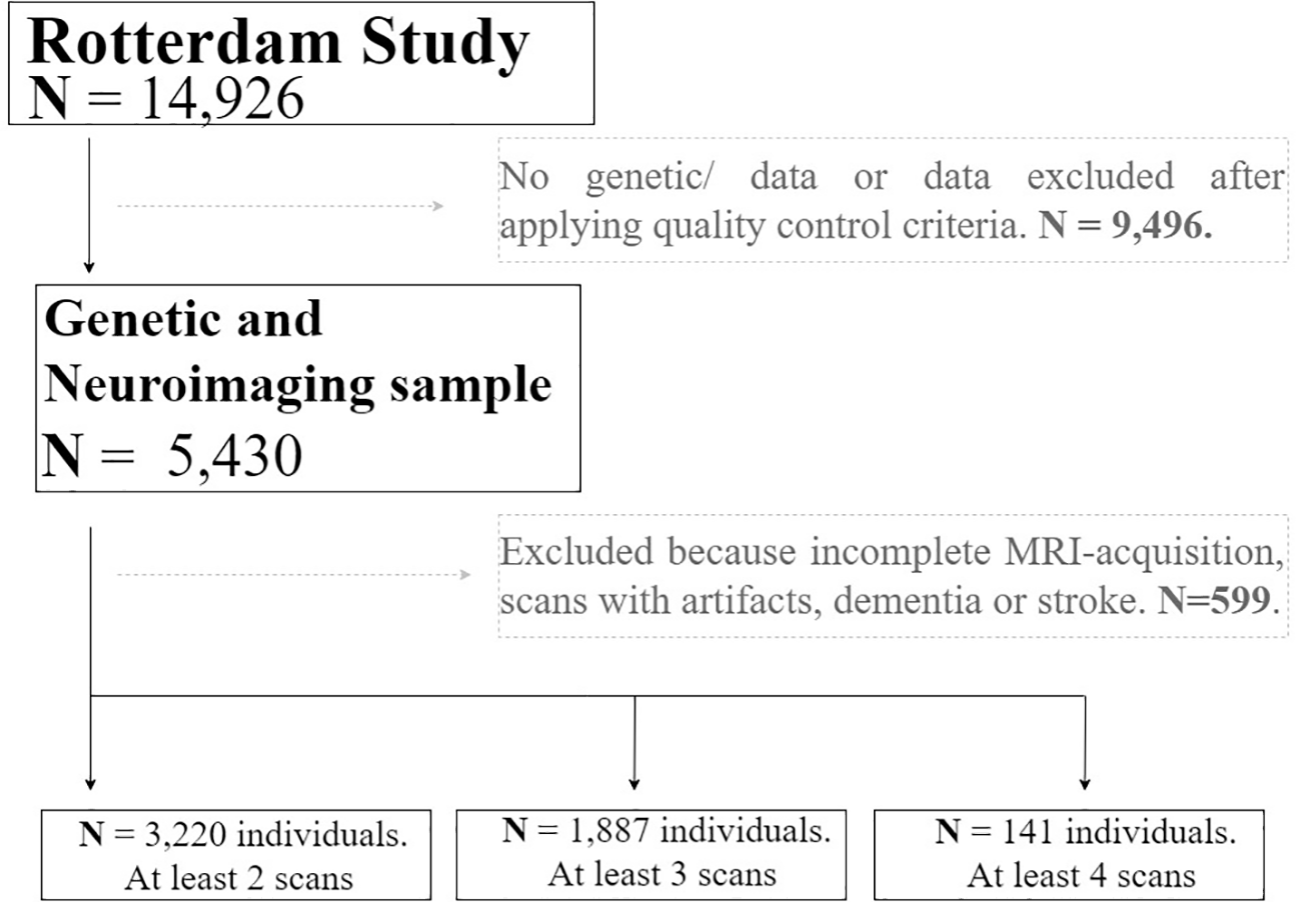
Flow chart depicting the final sample size of the real application. Solid lines and boxes represent individuals remaining in the study. Dashed lines and boxes represent individuals excluded. Reason and number of individuals excluded is indicated in dashed boxes. *Legend: N, size of the sample*.

We used mixed-effects models with random slopes and intercepts to calculate trajectories of volumetric MRI markers for each subject. The linear mixed models were fitted using the “lme” function within the R-package “nlme” (31). This model was selected based on previous brain trajectory assessment in the Rotterdam Study sample (32). We tested the longitudinal association between genome-wide genetic risk variants for ADHD and brain structures in fully adjusted models. A total of 3,220 individuals with at least two repeated measures of MRI-scan were included, resulting in 8,468 observations in total (**Figure 1**). Moreover, to account for possible non-linearity in brain structural trajectories, the age of the individuals at each measurement was used as the time variable of the model. Furthermore, after an exploratory analysis, splines of age with one *knot* were used in all models. Fixed effects of the model included: sex, and total intracranial volume (ICV). In addition, age-by-genotype interactions for each volume were included in the mixed-effects model to test whether age moderated genetic effects on longitudinal brain changes. The age-dependent model allows obtaining the difference in the change in average regional volume per additional year of age in spline, and the change in the slope for each spline, depending on genotypes effects. The coefficients of the interaction terms quantified the existence of possible slope differences of the trajectories explained by genotype.

#### Baseline Models

We used general linear models to test genetic influences on baseline differences in brain volumes. We also assessed age-dependent effects to test whether age moderated genetic effects on regional brain structures. Age-by-genotype interactions for each volume were included in the model. We used data corresponding to the first scan-acquisition of the whole sample (first observation per subject, *N*=3,220), as would be the case in a simple single observation cross-sectional study. As in the longitudinal models, splines of age with one knot were used in all models. Fixed effects included sex and ICV.

#### Post Hoc Analyses

In addition, we performed *post hoc* analyses to facilitate interpretation of the results. Among individuals 70 years of age or older (N=900, 2,084 observations), we tested whether regional brain volumes and/or longitudinal brain changes were associated with genetic effects or whether age moderated those genetic effects.

#### Multiple Comparison Correction

The weighted effects of baseline and longitudinal models were corrected for multiple comparisons. Since brain outcomes are correlated, we calculated the effective number of independent outcomes, *k_eff_*, using a permutation procedure, assuming 10,000 permutations. Additionally, we used the Bonferroni method for multiple testing correction. The threshold of significance was set following the formula $\frac{k_{eff}}{n_{SNPs}},$ were *k_eff_* represents the p-value threshold obtained through the permutation procedure, and *n_SNPs_* the number of genetic variants assessed in the models. The resulting adjusted threshold of significance was set to p < 2E-04. All statistical analyses were performed using R version 3.3.4.

## Results

### Descriptives

Descriptive characteristics of the subjects with at least two valid MRIs and descriptive of the brain volumetry for each scan acquisition are presented in **Table 1**. The study included 1,731 women and 1489 men between 50 and 100 years of age at baseline (65.3 **±** 9.3). The distribution in the percentage of women/men is balanced in all the scan acquisitions, while the age becomes slightly higher in the last visit, as expected. **Figure S1** shows the pattern of correlation (Pearson correlation statistics) among all brain structures included in the study. Further descriptive of brain structures included in the study can be found at **Supplemental Table 2**.

**Table 1:** Participant characteristics.

	N	Age (m at each scan ± SD; years)	Sex distribution (W/M)(%)	TIV (m ± SD; mm3)
***scan 2***	3,220	65.3(9.3)	1,731/1,489 (53.8%)	1,486,432 (161,510)
***scan 3***	1,887	64.5(7.3)	989/898 (52.4%)	1,491,226 (158,878)
***scan 4***	141	74.8(3.9)	59/82 (41.8%)	1,468,570 (174,479)

Mean (m) and standard deviation (SD) are shown for continous variables. TIV, total intracranial volume; W, women; M, men.

### Longitudinal Association Results

**Figure 2:**
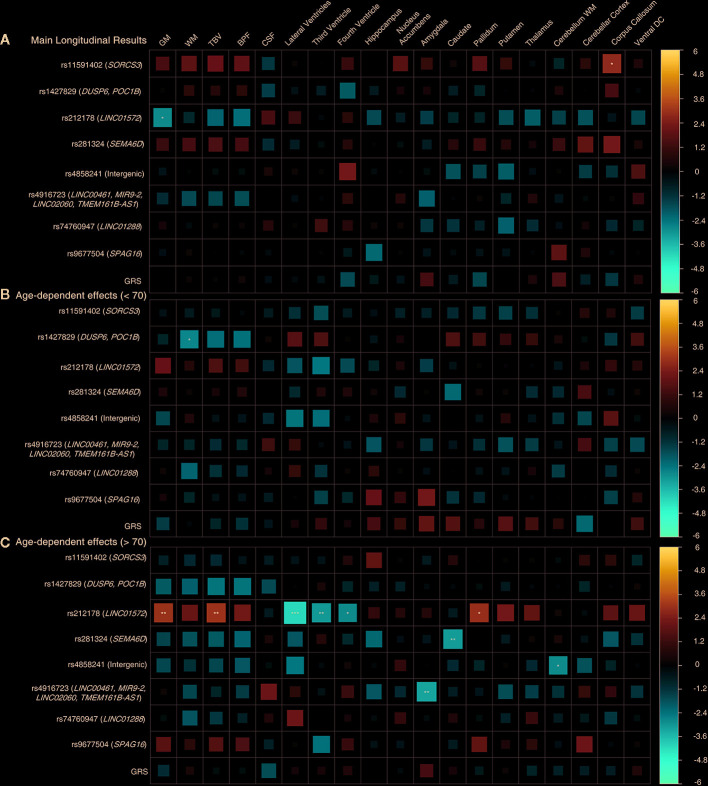
Longitudinal design whole sample. **(A)** Main genetic results. **(B)** Age-by-SNP results, spline 1. **(C)** Age-by-SNP results, spline 2***Pvalue = 0.0002 (Bonferroni correction); **Pvalue = 0.003 (BH correction); *Pvalue = 0.05 (nominal p-value). Color scale represents the normalized effect sizes. Legend: GM, Grey Matter; WM, White Matter; TBV, Total Brain Volume; BPF, Brain Parenchymal Fraction; CSF, Cerebrospinal Fluid; DC, Diencephalon; GRS, Genetic Risk Score.

**Figure 3:**
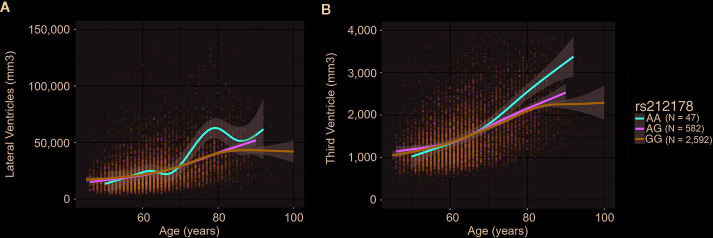
Trajectory differences of ventricle sizes between rs212178 genotypes In red, green and blue, the average trajectory of AA, AG, and GG genotypes, respectively. The x-axis represents the age at time of the scan (years), the left y-axis represents the value of the ventricle size (mm3): **(A)** *Lateral ventricles* **(B)** *Third ventricle*.

**Figure 4:**
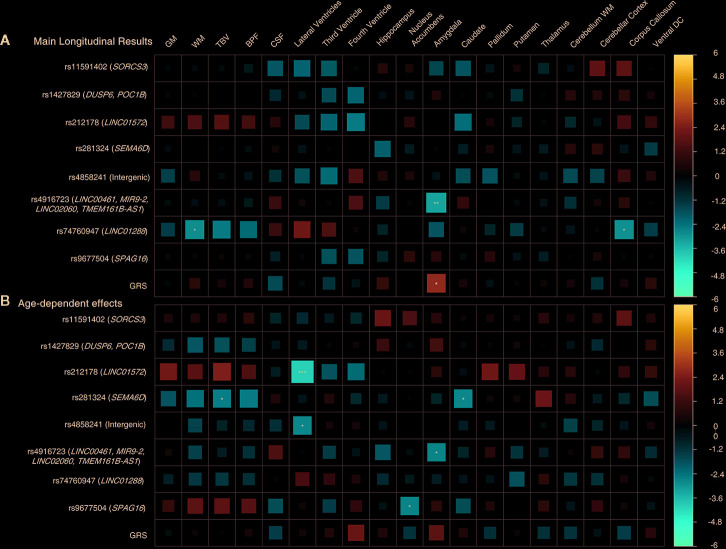
Longitudinal design, subset of individuals older than 70 years. **(A)** Main results. **(B)** Age-by-SNP results. ***Pvalue = 0.0002 (Bonferroni correction); **Pvalue = 0.003 (BH correction); *Pvalue = 0.05 (nominal p-value). Color scale represents the normalized effect sizes. Legend: GM, Grey Matter; WM, White Matter; TBV, Total Brain Volume; BPF, Brain Parenchymal Fraction; CSF, Cerebrospinal Fluid; DC, Diencephalon; GRS, Genetic Risk Score.

We observed significant age-dependent effect of the rs212178 (intron variant *LINC01572* at chromosome 16) in trajectories of ventricular size (lateral ventricles, P= 4E-05; third ventricle, P=2.5E-03; fourth ventricle, P=5.5E-03) (**Figures 2B, C** and **Supplemental Table 3**). Specifically, carriers of the protective ADHD allele G of rs212178 were associated with a less pronounced increase in lateral ventricle volumes among individuals 70 years of age or older (**Figure 3B**). Pointedly, among subjects > 70 years of age, we observed significant differences in trajectories (slope and intercept differences) for the different genotypes of rs212178 on ventricle size (lateral ventricles, P=7.3E-05) (**Figure 4** and **Supplemental Table 4**).

In addition, rs4916723 (intron variant *LINC00461* at chromosome 5), and rs281324 (intron variant *SEMA6D* at chromosome 15) displayed nominal significant age-dependent effects, which not survive multiple comparison, in trajectories of amygdala volume (P=1.4E-03) and caudate volume (P=1.8E-03), respectively.

No additional significant main effects were found after multiple comparisons correction (**Figures 2A** and **3A** and **Supplemental Tables 5** and **6**). Likewise, no significant effects were found for GRS.

### Baseline Association Results

**Figure 5:**
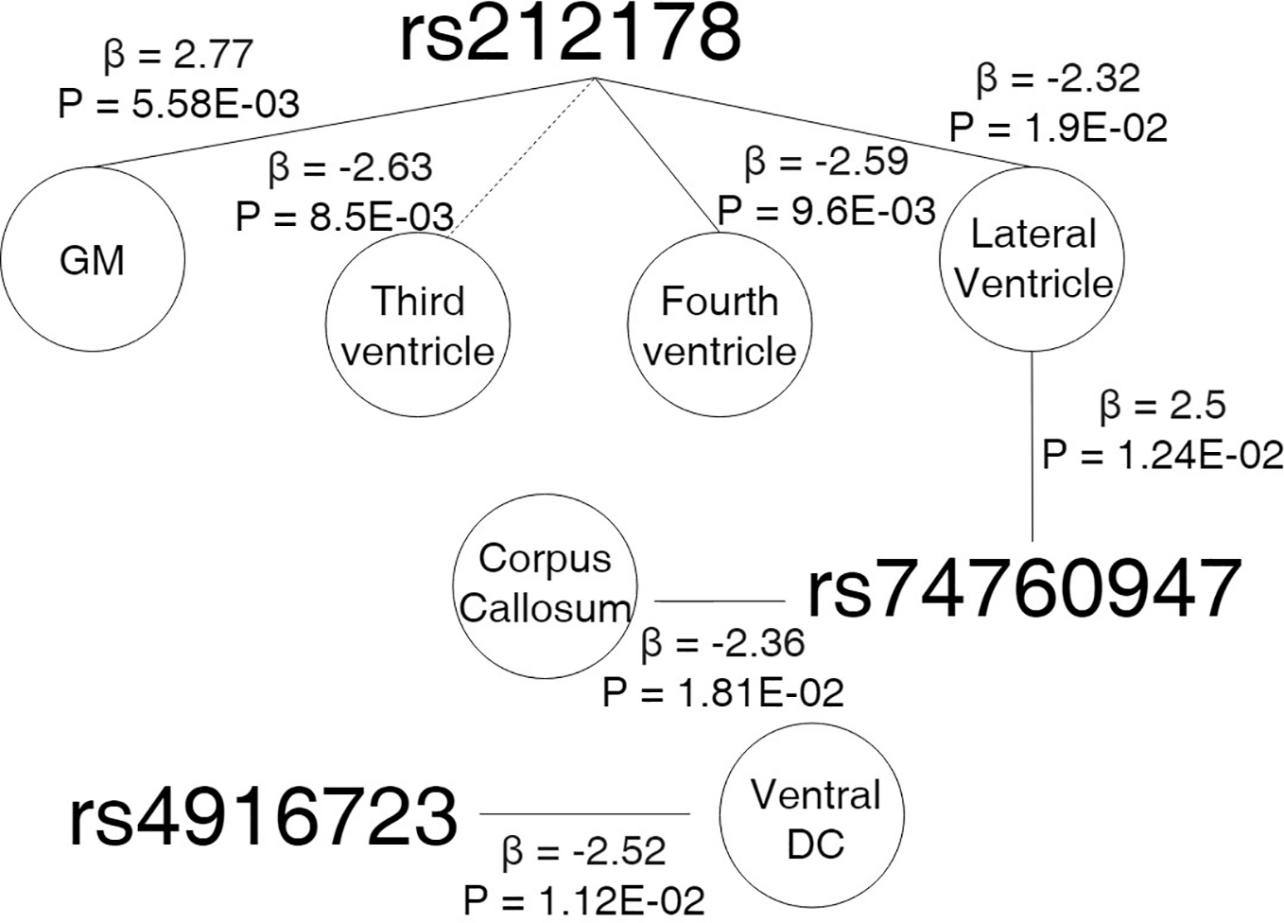
Cross sectional design. Age-by-genotype results. Straight lines represents a nominally significant age-by-SNP interaction at the first spline. Dotted lines represents a significant age-by-SNP interaction at the second spline. Parallel dotted lines represents a significant age-by-genotype interaction term in both splines. Legend: GM, Grey Matter; DC, Diencephalon; β, Effect size; P, p-value.

**Figure 5** showed results for the adjusted age-by-SNP interaction coefficients. None of the results remained significant after multiple comparison correction. Furthermore, no age-by-GRS interaction effects were found on either brain structure of interest.

Post hoc analyses suggested patterns of age-dependent effects of the rs212178 in ventricle size (lateral ventricles, P=0.01; and fourth ventricle, P=0.009) on individuals up to 70, sustained also among individuals 70 years of age or older (lateral ventricles, P=0.03; fourth ventricle, P=0.041: third ventricle, P=0.008), which reinforce our longitudinal results [**Supplemental Tables 7–10**].

## Discussion

According to our knowledge, this is the first study to investigate the effects of genome-wide significant SNPs for ADHD on longitudinal brain changes and its age-dependent effects in an adult population. Our main finding suggests that carriers of the minor allele G of rs212178 (chr16, *LINC01572*) were associated with a smaller increase of ventricular volume—indirectly reflecting lower brain atrophy—during aging, compared with homozygotes for the A allele. Interestingly, the G allele of rs212178 has been reported as a protective variant of ADHD (27) (OR=0.891; P=7.68×10-9), suggesting a relationship between the protective genetic effect on ADHD and less brain atrophy across the lifespan. Moreover, nominally significant age-dependent effects were identified, in which carriers of protective ADHD variants (C allele carriers of rs4916723 and rs281324) had smaller increases in the amygdala and caudate volumes.

However, these results should be interpreted considering its limitations, especially, due to the unavailability of a replication sample. First, in the present study, only a single variant was suggested affecting longitudinal changes on ventricle volumes with a small effect size. We can hypothesize a lack of statistical power in our analysis, but also the existence of a pleiotropic effect that could involve multiple different effects of genes to ADHD (20). Second, there are still many genetic variants contributing to the heritability of ADHD which remained undiscovered, and that therefore were not included in the models of the study. Third, we should be cautious because although ventricular size can represent accumulation of brain atrophy, it might also indirectly reflects a loss of brain tissue in other regions, which could confuse our results. Fourth, we should take into account that, in general, brain volume measurements are only a crude simplification of the complex anatomical brain changes, and often ignore the fact that longitudinal changes are not uniform across a brain structure. Finally, the study sample belongs to the general population; thus, the significant effects identified in this study do not necessarily imply a causal relationship with the clinical presentation of ADHD symptoms, but instead may represent a proxy for the potential causes that underlie their internal physiopathology.

Nevertheless, the present study identified intraindividual changes in brain structures using longitudinal data, which include at least two scanner acquisitions per individual. This provides a more valid measure of the brain structural change than extrapolating an estimate of change from separate individuals across a range of ages using cross-sectional designs. Furthermore, compared to the cross-sectional design, the longitudinal design can provide increased statistical power by reducing the confounding effect of between-subject variability and provide unique insights into the temporal dynamics of the underlying biological process of neurodevelopmental domains (33). Moreover, we considered the statistical interaction between genetic variations and age because of the implications for the shape of the distribution of onset age in risk analyses, which improves the understanding of the degree and direction of change over time (34, 35).

Progressive enlargement of the ventricular system is a common finding in several neurologic and psychiatric disorders including dementia (36), Parkinson’s disease (37), multiple sclerosis (38, 39), schizophrenia (40) and it has been extensively discussed in the context of brain cerebral atrophy and cognitive impairment (41). Interestingly, ventricular enlargement has been suggested as a neuroimaging-based biomarker in normal aging (42, 43), which may herald the cognitive decline associated with the onset and progression of Alzheimer’s disease (44–46). In this line, our results also suggested that age-related changes in G-allele carriers of rs212178 are important on prevention of brain atrophy during adulthood.

Although the rs212178 SNP was located within the *LINC01572*, fine-mapping results showed that the LD region was close to the protein coding zinc finger homeobox 3 (*ZFHX3*) gene. Thus, we cannot discard that this SNP may be responsible for regulation of the *ZFHX3* gene. This gene encodes cardio-enriched transcription factors, and regulates myogenic and neuronal differentiation (47). In addition, is highly expressed in human stem cell-derived cardio myoblasts and it has been reported as a one of the major atrial fibrillation (AF) susceptibility-conferring genes and an important regulatory factor which modifies circadian function (48–51). Indeed, several studies have reported ventricular enlargement associated with AF and other related cardiovascular phenotypes (52–54). This is in agreement with some other studies consistently suggesting that ventricular dysfunction and AF reduce cerebral blood flow exerting negative influences on cognitive function along in the aging process (55, 56). Hence, our findings might add to the current evidences relating similar genetic mechanisms of ventricular enlargement, aging, and adult ADHD through cardio metabolic pathways.

Finally, our results suggested the involvement of protective genetic ADHD factors and amygdala and caudate volume trajectories. These associations did not survive multiple testing correction, and therefore need to be replicated before further conclusions can be drawn. However, both brain regions have been well-described in the literature, and several meta-analyses have elucidated their involvement in ADHD (21, 57–59). Moreover, smaller caudate and amygdala volumes have been reported to be associated with cognitive deficits, the inhibition of attentional domains, and motor function constraints (60–63). Thus, our results may also suggest the existence of indirect causal effects on the biological mechanisms underlying the lifespan trajectories of ADHD symptoms, which may be mediated through impacts on brain structures.

To sum up, results obtained showed that specific effects of genetic variants associated with ADHD in adulthood are quite modest to elucidate longitudinal brain changes, but could suggest signs of brain atrophy. Such research furthers our understanding of the extent to which and how brain volume trajectories are genetically determined. Hence, research in imaging genetic field may greatly benefit from longitudinal designs, which represent a potential form to increase the statistical power to detect significant causal factors affecting structural brain changes.

## Data Availability Statement

Due to patient privacy, individual level genetic data cannot be made publicly available. Researchers who wish to use data of the Rotterdam Study must obtain approval from the Rotterdam Study Management Team. They are advised to contact the PI of the Rotterdam Study, MI (m.a.ikram@erasmusmc.nl).

## Ethics Statement

The studies involving human participants were reviewed and approved by The Rotterdam Study has been approved by the Medical Ethics Committee of the Erasmus MC and by the Ministry of Health, Welfare and Sport of Netherlands, implementing the Wet Bevolkingsonderzoek: ERGO (Population Studies Act: Rotterdam Study). The patients/participants provided their written informed consent to participate in this study.

## Author Contributions

NV-T and HA conceived the original idea of the study. NV-T performed the computations. EV contributed improving the R scripts and verified the analytical methods. NV-T wrote the manuscript in consultation with HA and MV. HA supervised the project. All authors contributed to the article and approved the submitted version.

## Funding

NV-T is funded by a post-doctoral grant, Juan de la Cierva Programme (FJC2018-038085-I), Ministerio de Ciencia, Innovación y Universidades – Spanish State Research Agency, and of a European Molecular Biology Organization (EMBO) Short-Term Fellowship (#8576). Her research has received additional support of “la Caixa” Foundation (LCF/PR/GN17/10300004) and the Health Department of the Catalan Government (Health Research and Innovation Strategic Plan (PERIS) 2016-2020 grant# SLT002/16/00201), the EU COST Action 15120 Open Multiscale Systems Medicine (OpenMultiMed) and Centro de Investigación Biomédica en Red de Epidemiología y Salud Pública (CIBERESP). HA was supported by ZonMW grant numbers 916.19.15 and 916.19.151.

The generation and management of GWAS genotype data for the Rotterdam Study are supported by Netherlands Organization of Scientific Research NWO Investments (no. 175.010.2005.011, 911-03-012). This study is funded by the Research Institute for Diseases in the Elderly (014-93-015; RIDE2), Netherlands Genomics Initiative (NGI)/Netherlands Organization for Scientific Research (NWO) project no. 050-060-810. All CRG authors acknowledge the support of the Spanish Ministry of Science, Innovation and Universities to the EMBL partnership, the Centro de Excelencia Severo Ochoa and the CERCA Programme/Generalitat de Catalunya.

## Conflict of Interest

The authors declare that the research was conducted in the absence of any commercial or financial relationships that could be construed as a potential conflict of interest.
