# Supplementary material for: Aging-Dependent Genetic Effects Associated to ADHD Predict Longitudinal Changes of Ventricular Volumes in Adulthood
Source: Front Psychiatry. 2020 Jun 29;11:574. doi: 10.3389/fpsyt.2020.00574 (PMC7344235; doi:10.3389/fpsyt.2020.00574)
Supplement: Supplementary file 1 [file DataSheet_1.zip › Tables_manuscript/ST1.docx]

**Table S1.** Full summary statistics for all SNPs tested. SNPs are LD independent (r2 < 0.1), and are merged into one locus when located with a distance less than 400kb. The location (chromosome [Chr] and base position [BP]), alleles (A1, A2), allele frequency (A1 freq), odds ratio (OR) of the effect with respect to A1, and association P-value of the index variant are given, along with genes within 50kb of the credible set for the locus.

| **SNP** | **CHR** | **BP** | **A1** | **A2** | **A1 freq** | **OR** | **P-value** | **Gene** |
| --- | --- | --- | --- | --- | --- | --- | --- | --- |
| rs9677504 | 2 | 215,181,889 | G | A | 0.109 | 1.124 | 1.39x${10}^{-8}$ | *SPAG16* |
| rs4858241 | 3 | 20,669,071 | T | G | 0.622 | 1.082 | 1.74x${10}^{-8}$ | *Intergenic* |
| rs4916723 | 5 | 87,854,395 | A | C | 0.573 | 0.926 | 1.58x${10}^{-8}$ | *LINC00461, MIR9-2, LINC02060, TMEM161B-AS1* |
| rs74760947 | 8 | 34,352,610 | A | G | 0.957 | 0.835 | 1.35x${10}^{-8}$ | *LINC01288* |
| rs11591402 | 10 | 106,747,354 | T | A | 0.224 | 0.911 | 1.34x${10}^{-8}$ | *SORCS3* |
| rs1427829 | 12 | 89,760,744 | A | G | 0.434 | 1.083 | 1.82x${10}^{-9}$ | *DUSP6, POC1B* |
| rs281324 | 15 | 47,754,018 | C | T | 0.531 | 0.928 | 2.62x${10}^{-8}$ | *SEMA6D* |
| rs212178 | 16 | 72,578,131 | A | G | 0.883 | 0.891 | 7.68x${10}^{-9}$ | *LINC01572* |
