# Supplementary material for: Aging-Dependent Genetic Effects Associated to ADHD Predict Longitudinal Changes of Ventricular Volumes in Adulthood
Source: Front Psychiatry. 2020 Jun 29;11:574. doi: 10.3389/fpsyt.2020.00574 (PMC7344235; doi:10.3389/fpsyt.2020.00574)
Supplement: Supplementary file 1 [file DataSheet_1.zip › Tables_manuscript/ST2.docx]

**Table S2.** Full summary descriptive of brain structures included in the study. Means, standard deviations (SD), Median, and ranges values are shown.

|  | *scan 1; N=4,831* | | | |  | *scan 2; N=3,220* | | | |
| --- | --- | --- | --- | --- | --- | --- | --- | --- | --- |
| ***Variable*** | **Mean(SD)** | **Min** | **Median** | **Max** |  | **Mean(SD)** | **Min** | **Median** | **Max** |
| Amygdala, mm3 | 2,713 (393) | 1.045 | 2.709 | 4.448 |  | 2750.6(389.04) | 1.032 | 2.749 | 4.857 |
| Caudate Nucleus, mm3 | 6,896 (1,099) | 3.454 | 6.745 | 14.912 |  | 6859.1(1077.93) | 4.453 | 6.724 | 13.598 |
| Cerebellum Cortex, mm3 | 99,129(11,068) | 34.039 | 98.882 | 148.289 |  | 99918.4(11039.07) | 58.138 | 99.575 | 143.131 |
| Cerebellum WM, mm3 | 23,679 (3,517) | 6.398 | 23.476 | 44.160 |  | 23759.2(3466.06) | 13.135 | 23.502 | 49.391 |
| Corpus callosum, mm3 | 2,625 (548) | 370 | 2.652 | 4.601 |  | 2628.2(541.04) | 643 | 2.643 | 4.521 |
| Fourth Ventricle, mm3 | 1,996 (576) | 218 | 1.907 | 7.865 |  | 2035.9(590.22) | 734 | 1.954 | 8.967 |
| Globus Pallidus, mm3 | 2,895 (471) | 1.377 | 2.870 | 6.318 |  | 2886.1(449.35) | 1.427 | 2.876 | 5.367 |
| Hippocampus, mm3 | 7,707 (1,079) | 2.191 | 7.793 | 11.722 |  | 7833.1(1071.05) | 3.117 | 7.932 | 11.277 |
| Lateral Ventricles, mm3 | 26,464 (16,708) | 2.900 | 21.832 | 146.082 |  | 26785(16869.89) | 3.908 | 21.996 | 162.490 |
| Nucleus Accumbens, mm3 | 1,049 (176) | 264 | 1.042 | 2.159 |  | 1051.5(173.97) | 507 | 1.042 | 1.885 |
| Putamen, mm3 | 9,102 (1,280) | 3.965 | 9.019 | 17.775 |  | 9103.1(1230.39) | 5.123 | 9.061 | 20.864 |
| Thalamus, mm3 | 12,554 (1,567) | 7.178 | 12.443 | 26.764 |  | 12562.8(1524.85) | 8.175 | 12.490 | 29.273 |
| Third Ventricle, mm3 | 1,551 (656) | 308 | 1.403 | 4.822 |  | 1580.5(660.05) | 128 | 1.430 | 5.189 |
| Ventral DC, mm3 | 7679(884) | 4.881 | 7.631 | 11.744 |  | 7777(881) | 4.949 | 7.750 | 11.771 |
| ICV, mm3 | 1,479,696 (161,628) | 926.412 | 1.475.734 | 2.075.800 |  | 1486431.8(161509.86) | 961.537 | 1.483.578 | 2.105.942 |
| GM, mm3 | 596,235 (58,456) | 396.242 | 593.973 | 802.581 |  | 598063.5(57164.82) | 406.333 | 595.949 | 795.062 |
| WM, mm3 | 434,979 (64,998) | 204.521 | 434.070 | 717.608 |  | 435958.6(62887.63) | 190.808 | 435.316 | 656.974 |
| TBV, mm3 | 1,031,215 (118,976) | 640.449 | 1.028.389 | 1.510.603 |  | 1034022.1(115598.59) | 684.065 | 1.030.530 | 1.434.771 |
| BPF, % | 0.7 (0.05) | 0 | 1 | 1 |  | 0.7(0.05) | 0 | 1 | 1 |
| CSF, mm3 | 1,266 (367) | 482 | 1.196 | 4.303 |  | 1286.7(356.69) | 571 | 1.218 | 3.819 |

|  | *scan 3; N=1,887* | | | |  | *scan 4; N=141* | | | |
| --- | --- | --- | --- | --- | --- | --- | --- | --- | --- |
| ***Variable*** | **Mean(SD)** | **Min** | **Median** | **Max** |  | **Mean(SD)** | **Min** | **Median** | **Max** |
| Amygdala, mm3 | 2759.1(374.07) | 1492 | 2751 | 4232 |  | 2602.6(417.55) | 1.384 | 2.575 | 3.645 |
| Caudate Nucleus, mm3 | 6805.4(1018.54) | 4299 | 6667 | 11463 |  | 6875.5(1221.42) | 4.901 | 6.660 | 12.620 |
| Cerebellum Cortex, mm3 | 100763.9(10792.79) | 70256 | 99930 | 138059 |  | 96941(10020.02) | 73.563 | 97.483 | 121.866 |
| Cerebellum WM, mm3 | 23978.7(3446.2) | 12854 | 23695 | 41765 |  | 21993(2842.77) | 13.123 | 21.882 | 30.297 |
| Corpus callosum, mm3 | 2651(521.21) | 624 | 2653 | 4882 |  | 2343.6(557.24) | 747 | 2.383 | 3.811 |
| Fourth Ventricle, mm3 | 2042.5(592.75) | 842 | 1964 | 9231 |  | 2121.9(866.68) | 932 | 2.006 | 9.732 |
| Globus Pallidus, mm3 | 2943.9(457.94) | 1629 | 2922 | 4974 |  | 2702.1(384.8) | 1.813 | 2.694 | 3.790 |
| Hippocampus, mm3 | 7922.4(1040.71) | 3731 | 7994 | 11218 |  | 7237.3(1034.33) | 4.190 | 7.359 | 9.758 |
| Lateral Ventricles, mm3 | 25662.2(15796.38) | 4623 | 21580 | 172963 |  | 36200.9(21934.68) | 9.002 | 30.541 | 195.491 |
| Nucleus Accumbens, mm3 | 1059.3(173.65) | 391 | 1049 | 1705 |  | 1005.3(164.91) | 651 | 998 | 1.640 |
| Putamen, mm3 | 9158.7(1220.54) | 5274 | 9064 | 15568 |  | 8621.4(1097.71) | 6.377 | 8.443 | 11.104 |
| Thalamus, mm3 | 12685.8(1542.15) | 7999 | 12625 | 33813 |  | 11851(1306.25) | 8.767 | 11.750 | 16.874 |
| Third Ventricle, mm3 | 1533(597.54) | 376 | 1396 | 5320 |  | 1977.8(744.44) | 814 | 1.835 | 5.629 |
| Ventral DC, mm3 | 7854(873) | 5092 | 7818 | 13968 |  | 7429(767) | 5.494 | 7.453 | 9.543 |
| ICV, mm3 | 1491225.6(158877.95) | 991952 | 1483989 | 2104746 |  | 1468569.5(174478.89) | 1.009.685 | 1.463.542 | 1.946.649 |
| GM, mm3 | 601487.7(55335.99) | 414281 | 599142 | 782188 |  | 574293.4(49750.73) | 445.626 | 572.779 | 717.230 |
| WM, mm3 | 439350.2(58896.54) | 238022 | 438587 | 646887 |  | 406666.3(58138.18) | 262.422 | 404.217 | 593.625 |
| TBV, mm3 | 1040837.9(109658.07) | 652303 | 1037746 | 1388786 |  | 980959.7(102646.47) | 745.082 | 974.872 | 1.282.747 |
| BPF, % | 0.7(0.04) | 0 | 1 | 1 |  | 0.7(0.04) | 1 | 1 | 1 |
| CSF, mm3 | 1287.2(351.46) | 583 | 1221 | 4911 |  | 1431.2(451.56) | 766 | 1.341 | 4.569 |
