# Supplementary material for: Aging-Dependent Genetic Effects Associated to ADHD Predict Longitudinal Changes of Ventricular Volumes in Adulthood
Source: Front Psychiatry. 2020 Jun 29;11:574. doi: 10.3389/fpsyt.2020.00574 (PMC7344235; doi:10.3389/fpsyt.2020.00574)
Supplement: Supplementary file 2 [file DataSheet_2.pdf]

Supplementary Material

Aging-dependent genetic effects associated to ADHD predict longitudinal changes of ventricular volumes in adulthood

1 Supplementary Tables and Figures

1.1 Supplementary Tables

Table S1. Full summary statistics for all SNPs tested. SNPs are LD independent ($r2 < 0.1$), and are merged into one locus when located with a distance less than 400kb. The location (chromosome [Chr] and base position [BP]), alleles (A1, A2), allele frequency (A1 freq), odds ratio (OR) of the effect with respect to A1, and association P-value of the index variant are given, along with genes within 50kb of the credible set for the locus.

Table S2. Full summary descriptive of brain structures included in the study. Means, standard deviations (SD), Median, and ranges values are shown.

Table S3. Results longitudinal design. Model: SNP*Age. Sample: All sample (N=3,200 individuals, 8,468 observations). Date and time of computation, outcome, model adjusted, as well as, effects (Beta1, Beta2), standard deviation (SE1, SE2), T-statistics (T1, T2) and p-values (P1, P2) are shown for each spline.

Table S4. Results longitudinal design. Model: Genetic main effects. Sample: All sample (N=3,200 individuals, 8,468 observations). Date and time of computation, outcome, model adjusted, as well as, effect (Beta), standard deviation (SE), T-statistic (T) and p-value (P) are shown.

Table S5. Results longitudinal design. Model: SNP*Age. Sample: ≥70 years old (N=900 individuals, 2,084 observations). Date and time of computation, outcome, model adjusted, as well as, effect (Beta), standard deviation (SE), T-statistic (T) and p-value (P) are shown.

Table S6. Results longitudinal design. Model: Genetic main effects. Sample: ≥70 years old (N=900 individuals, 2,084 observations). Date and time of computation, outcome, model adjusted, as well as, effect (Beta), standard deviation (SE), T-statistic (T) and p-value (P) are shown.

Table S7. Results cross-sectional design. Model: SNP*Age. Sample: All sample (N=3,200 individuals). Date and time of computation, outcome, model adjusted, as well as, effects (Beta1, Beta2), standard deviation (SE1, SE2), T-statistics (T1, T2) and p-values (P1, P2) are shown for each spline.

Table S8. Results cross-sectional design. Model: Genetic main effects. Sample: All sample (N=3,200 individuals). Date and time of computation, outcome, model adjusted, as well as, effect (Beta), standard deviation (SE), T-statistic (T) and p-value (P) are shown.

Table S9. Results cross-sectional design. Model: SNP*Age. Sample: ≥70 years old (N=900 individuals). Date and time of computation, outcome, model adjusted, as well as, effect (Beta), standard deviation (SE), T-statistic (T) and p-value (P) are shown.

Table S10. Results cross-sectional design. Model: Genetic main effects. Sample: ≥70 years old (N=900 individuals). Date and time of computation, outcome, model adjusted, as well as, effect (Beta), standard deviation (SE), T-statistic (T) and p-value (P) are shown.

1.2 Supplementary Figures

Supplementary Figure 1. Heatmap showing pair correlations across brain structures, with blue color indicating positive correlations and red color indicating negative correlations.
