# Supplementary figures and images for: Aging-Dependent Genetic Effects Associated to ADHD Predict Longitudinal Changes of Ventricular Volumes in Adulthood
Source: Front Psychiatry. 2020 Jun 29;11:574. doi: 10.3389/fpsyt.2020.00574 (PMC7344235; doi:10.3389/fpsyt.2020.00574)

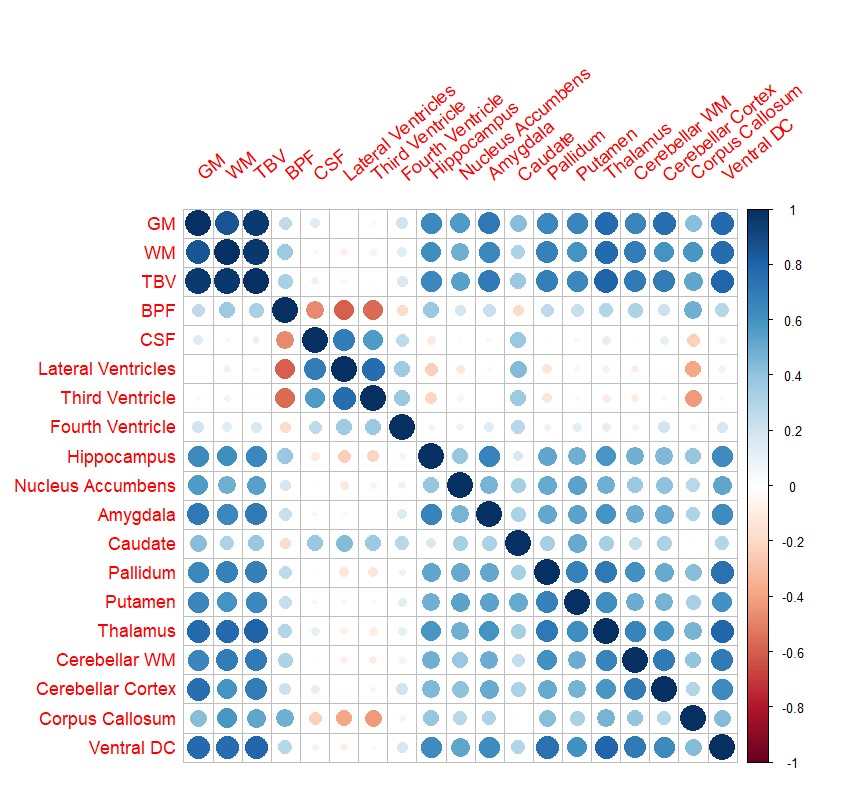

Supplement: Supplementary file 3 [file Image_1.jpeg]
